# Machine learning prediction of blood alcohol concentration: a digital signature of smart-breathalyzer behavior

**DOI:** 10.1038/s41746-021-00441-4

**Published:** 2021-04-20

**Authors:** Kirstin Aschbacher, Christian S. Hendershot, Geoffrey Tison, Judith A. Hahn, Robert Avram, Jeffrey E. Olgin, Gregory M. Marcus

**Affiliations:** 1 Division of Cardiology, Department of Medicine, University of California, San Francisco, San Francisco, CA USA; 2 Department of Psychiatry, Weill Institute for Neurosciences, University of California, San Francisco, San Francisco, CA USA; 3 Department of Psychiatry, University of North Carolina at Chapel Hill, Chapel Hill, NC USA; 4 Division of HIV, Infectious Diseases, and Global Medicine; Department of Medicine, University of California, San Francisco, San Francisco, CA USA

**Keywords:** Biotechnology, Computational biology and bioinformatics

## Abstract

Excess alcohol use is an important determinant of death and disability. Machine learning (ML)-driven interventions leveraging smart-breathalyzer data may help reduce these harms. We developed a digital phenotype of long-term smart-breathalyzer behavior to predict individuals’ breath alcohol concentration (BrAC) levels trained on data from a smart breathalyzer. We analyzed roughly one million datapoints from 33,452 users of a commercial smart-breathalyzer device, collected between 2013 and 2017. For validation, we analyzed the associations between state-level observed smart-breathalyzer BrAC levels and impaired-driving motor vehicle death rates. Behavioral, geolocation-based, and time-series-derived features were fed to an ML algorithm using training (70% of the cohort), development (10% of the cohort), and test (20% of the cohort) sets to predict the likelihood of a BrAC exceeding the legal driving limit (0.08 g/dL). States with higher average BrAC levels had significantly higher alcohol-related driving death rates, adjusted for the number of users per state *B* (SE) = 91.38 (15.16), *p* < 0.01. In the independent test set, the ML algorithm predicted the likelihood of a given user-initiated BrAC sample exceeding BrAC ≥ 0.08 g/dL, with an area under the curve (AUC) of 85%. Highly predictive features included users’ prior BrAC trends, subjective estimation of their BrAC (or AUC = 82% without the self-estimate), engagement and self-monitoring, time since the last measure, and hour of the day. In conclusion, an ML algorithm successfully quantified a digital phenotype of behavior, predicting naturalistic BrAC levels exceeding 0.08 g/dL (a threshold associated with alcohol-related harm) with good discrimination capability. This result establishes a foundation for future research on precision behavioral medicine digital health interventions using smart breathalyzers and passive monitoring approaches.

## Introduction

According to the World Health Organization, harmful use of alcohol accounts for 5% of the global disease burden, or 1 in 20 deaths^1^. This excess mortality arises both from behavioral sequelae (motor vehicle accidents, suicide, and interpersonal violence) and from medical morbidity (e.g., liver cirrhosis, cancers, pancreatitis, and psychiatric comorbidities)^2,3^. Smart breathalyzers are small hand-held devices that collect user-initiated voluntary readings, which have been available commercially since 2013. These devices reliably infer blood alcohol concentrations (BACs) from exhaled breath and integrate with a smartphone via a mobile application (app) and Bluetooth technology. Such smart breathalyzers could inform novel interventions to promote alcohol use behavior change. Furthermore, digital platforms providing real-time feedback could enable the opportunity to message users in critical moments and offer machine learning (ML)-driven, personalized “Just-in-Time” alcohol interventions^4^. To enable real-time intervention, an ML model would need to be capable of predicting future BAC risk thresholds with reasonably high sensitivity and specificity based on minimal information. Hence, we sought to investigate whether breath alcohol concentration (BrAC) levels associated with alcohol-related harms (BrAC ≥ 0.08 g/dL)^5^ can be predicted with reasonable accuracy in a large, international sample of smart-breathalyzer users, given behavioral, geolocation, and temporal data related to device and app usage.

Prior studies have examined the potential utility of personal breathalyzers for self-monitoring alcohol use in clinical and naturalistic settings^6^. Some commercially available smart breathalyzers have validity comparable to a police-grade device^7^. Although data generated by smart breathalyzers could be informative for predictive modeling, no studies have addressed this question. To evaluate real-world predictive potential, BrAC levels need to be collected under real-world conditions with naturalistic signal-to-noise profiles, representing typical user and technological sources of variability.

Naturalistic data from personal breathalyzers are, by definition, obtained during user-initiated drinking episodes. Despite this limitation, these data might inform the development of ML-based interventions targeting harm-reduction approaches (e.g., predicting those drinking episodes that are more likely to result in higher BrAC). These interventions can complement other ML-based algorithms that might attempt to anticipate the onset of a drinking episode. Moreover, to the extent that routine BrAC feedback can provide corrective information regarding perceived versus actual intoxication, continued personal breathalyzer use could constitute an intervention in itself. This possibility is consistent with empirical support for BrAC discrimination training, in which drinkers are taught to accurately estimate their BrAC and recognize when BrAC is approaching risky levels^8^. At least one smart-breathalyzer app (e.g., BACtrack) asks users for subjective, self-estimates of their BrAC prior to displaying objective BrAC results, presenting a possible opportunity to study BrAC discrimination naturalistically. To date, no study has examined predictors of heavy drinking using objective, naturalistic samples of BrAC measurements—or examined temporal changes in actual versus perceived intoxication—at a large scale. To establish a foundation for digital health interventions in this area, this study leverages a unique dataset with nearly one million BrAC observations to validate a predictive algorithm of high BrAC and to examine temporal changes in perceived versus measured intoxication.

ML approaches are well suited to leverage large volumes of data, such as those generated by smart breathalyzers, and have exhibited strong performance in a variety of health-related applications^9^. One particular class of ML algorithms, called ensemble tree algorithms^10^, offers several advantages in this case, such as the ability to easily model nonlinearities, interactions, and incorporate missing data as predictive features, as well as the capacity to easily parallelize and scale^11^. The ability to identify the important features that explain the ML model can assist in determining the most impactful modifiable targets.

Digital phenotyping of behavior constitutes a new frontier in behavioral medicine^12,13^. However, very little is known about the naturalistic patterns of commercial smart-breathalyzer use and their association with population-based health outcomes, such as intoxicated driving-related mortality rates. Expert recommendations to reduce alcohol consumption via smartphone apps advocate self-monitoring^14^. Moreover, research shows that the more users underestimate their level of intoxication, the greater their likelihood of driving after drinking^15^. Hence, asking users to provide a *self-estimation* of intoxication in the app, before they receive their objective results from the smart breathalyzer, might augment the benefits of self-monitoring. This study is the first to investigate whether continued self-monitoring (by self-estimations and device use) does indeed result in reduced smart-breathalyzer-measured blood alcohol levels over time in a large cohort of smart-breathalyzer users.

We sought to apply an ML algorithm to smart-breathalyzer data, obtained from a multinational cohort and roughly one million observations, in order to predict elevated BrAC levels (≥0.08 g/dl). As an indicator of product value, we quantified the extent to which this ML algorithm using smart-breathalyzer data could predict objectively high BrAC levels, *above and beyond a user’s subjective BrAC estimate*. Next, we describe the broader patterns of use and provide explainability analyses of the most important predictive features. To guide future smart-breathalyzer-based interventions, we test whether repeated use of the App’s BrAC self-estimation feature acts to improve user’s BrAC discrimination capability over time, and utilize the algorithm’s results to highlight where targeted messaging might be useful. Finally, we show the external validity of these measurements by demonstrating associations with alcohol-related motor vehicle deaths across the United States. To our knowledge, this is the first and only study of naturalistic, population-based BrAC data recorded in real time during drinking events.

## Results

### Characterization

A total of 973,264 user-initiated BrAC recordings were obtained from 33,452 users, who used a BACtrack device for a median of 3.5 (interquartile range (IQR): 1.5–12.9) months (Supplementary Figure 1) (see “Methods” for further details). Supplementary Tables 1–3 provide counts of the number of distinct users and recordings for each country and each state in the United States, as well as the break down by year. Roughly half (52%) of recordings were taken in the United States, and an additional 35% could not be assigned to a country. The mean BrAC across all recordings was 0.057 ± 0.065 g/dL and the within-user aggregated BrAC mean was 0.059 ± 0.042 g/dL. Analysis of state-level data revealed mean BrAC values ranging from 0.035 g/dL in Utah to 0.133 g/dL in Montana (Supplementary Figure 3).

Reflecting the overall quantity and frequency of engagement, the median (IQR) number of BrAC recordings taken per user was 80 (31–207), and the median (IQR) number of days on which at least one BrAC recording was taken was 27 (10–68). On average, users logged 2.69 ± 2.21 BrAC measurements per day the device was used, over a median use duration of 106 (45–388). User-entered app data for the number of drinks consumed had significant missing data (see “Methods”) and provided little useful variance, with the median number being one drink entered (1–1) and the 95% percentile corresponding to two drinks entered.

**Fig. 1:**
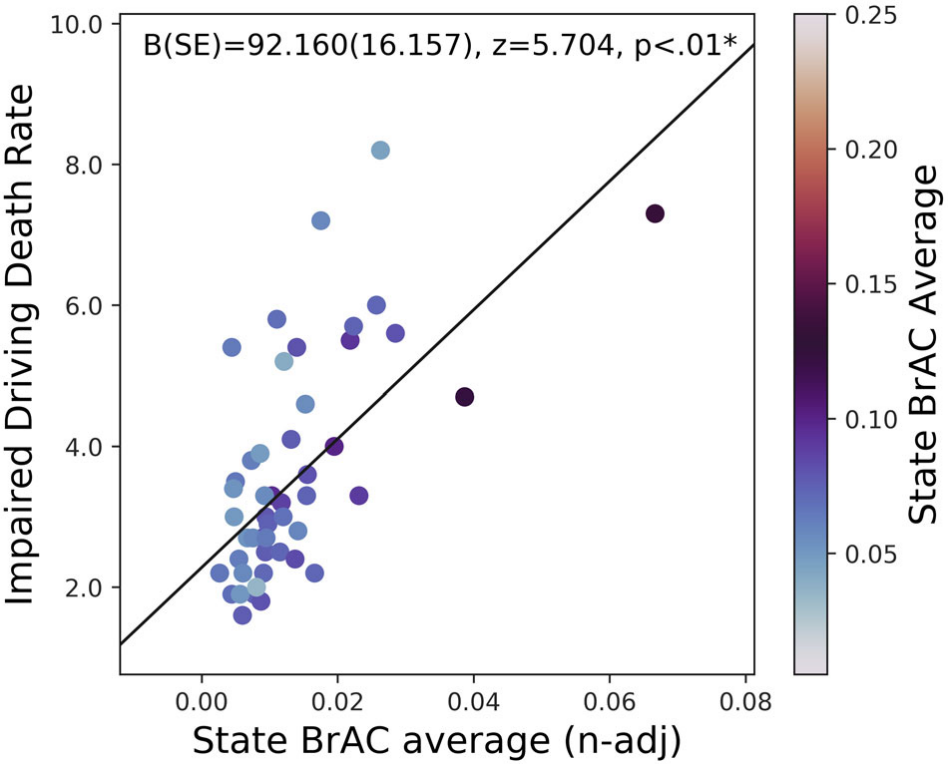
Association of average BrAC levels and motor vehicle death rates by state. States with higher BrAC levels on average tend to also have higher rates of death (per 100,000 population) for people killed in crashes involving a driver with BrAC ≥ 0.08 g/dL, when state average BrAC levels are adjusted for the number of users per state. This scatterplot represents a linear regression model (using robust fit), which depicts the significant association *p* < 0.01) between each state’s motor vehicle death rate and the average BrAC level, adjusted by the number of users. All data are from the year 2014; impaired-driving data are from the Centers for Disease Control and Prevention.

We investigated whether naturalistic BrAC data from the individuals in our particular cohort may reflect broader population-level behaviors relevant to alcohol-related health risks, using a subset of the data from 2014, which aligned with publicly available data (see “Methods”). Specifically, we tested whether states in the United States exhibiting higher user-initiated smart-breathalyzer BrAC levels had higher impaired-driving death rates. In regression analysis among 53,674 BrAC observations from 2641 distinct users, we observed a significant association between higher average BrAC levels within our cohort and higher motor vehicle death rates (*B* = 92.160 (95% confidence interval (CI) = 60.493–123.826), *z* = 5.704, *p* < 0.001; Fig. 1). Supplementary Figure 2 (view by the web browser) depicts a dynamic map of the United States, which allows the reader to toggle the view between state motor vehicle death rates and state average BrAC levels by clicking on the image background.

### BrAC prediction

Per standard ML convention, users were randomly assigned to train, validation, and test sets using a 70–10–20 percentage split. Forty-six features (Supplementary Table 4) were entered into a stochastic gradient boosting classification tree (GBCT) model (learning rate = 0.1, n-estimators = 89, L1 regularization = 0.5, L2 regularization = 0.5) to predict high (≥0.08) versus low (<0.08) BrAC levels. As the ratio of low to high BrACs was 2.37, all models were trained using built-in class balancing functions, which place a differential penalty in the cost function, thereby remediating the tendency for the model to primarily learn the more prevalent class (i.e., low BrAC values.) A validation set was used to tune the hyperparameter settings. Predictions were made in an independent test set of new randomly selected users among those not previously included in the model.

**Fig. 2:**
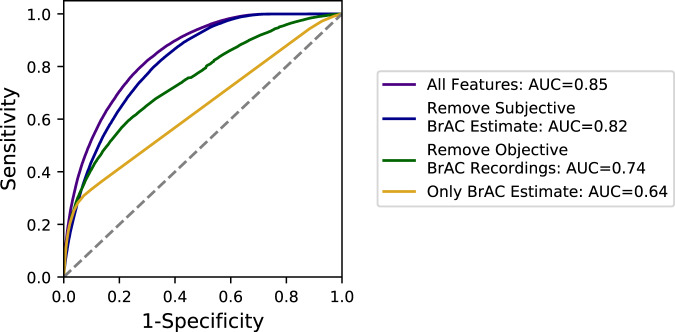
Final model performance evaluation. To quantify the value of various data sources derived from the smart-breathalyzer device and app, we contrasted the full model with all 46 features to other nested GBCT models, in which we removed different feature(s) (also see Table 1). To benchmark the success of these models, we quantified a base model, which used the user’s subjective BrAC estimate as the sole predictor of the subsequently measured objective smart-breathalyzer BrAC levels. All model parameters were derived from the training set, and evaluated in a separate test set (20%) of randomly selected users not seen by the training set. The full model with all features (purple line) performed substantially better (21% higher; AUC = 0.85 vs 0.64) than the base model with only the user’s self-reported BrAC Estimate only (gold line). Removal of the BrAC estimate from the full feature set reduced the AUC by only 3% (blue line), whereas removal of all features derived from prior objective BrAC recordings reduced the AUC by 11% (green line), thereby demonstrating the importance of prior smart-breathalyzer time-series measures for this algorithm’s performance. ROC-AUC receiver-operating characteristic area under the curve.

Smart-breathalyzer users have the option to record their own subjective BrAC estimate in the app before breathing into the device. We used the user’s subjective BrAC estimate to predict high or low smart-breathalyzer BrAC measurements as a baseline GBCT model for comparison, against which the models with varying feature sets should be contrasted. We additionally included a majority class model for comparison, which performed no better than random. The area under the curve (AUC) for this base model was 0.64 in a randomly selected, separate test set of users. In contrast to the base model, the final GBCT model was yielded a substantially higher AUC of 0.85, sensitivity of 0.80, and specificity of 0.73 (Fig. 2 and Table 1). Furthermore, the model continued to perform well (AUC = 0.82), when removing the user’s subjective BrAC Estimate, but demonstrated a more substantial decrease (AUC = 0.74) when removing all features derived from the history of prior BrAC measurements, gathered from the smart-breathalyzer device.

**Table 1:** Model performance characteristics and comparison.

Model	ROC-AUC	Accuracy	*F*1	Sensitivity	Specificity	Precision
1. All features	0.85	0.75	0.76	0.80	0.73	0.55
2. All features except the subjective BrAC estimate	0.82	0.71	0.72	0.80	0.67	0.50
3. All features except the prior objective BrAC recordings	0.74	0.72	0.72	0.59	0.77	0.52
4. Only the BrAC estimate	0.64	0.75	0.71	0.31	0.93	0.64
5. Majority class	0.50	0.71	0.59	0.00	1.00	Undefined

We compared a series of gradient boosted classification tree (GBCT) models, in order to evaluate algorithmic performance and understand how specific data sources impacted model performance. We contrasted the full model performance using all 46 features (Model 1) with performance after removing the user’s subjective BrAC estimation (Model 2) or after removing all the features derived from smart-breathalyzer BrAC measurements (Model 3). Model 3, therefore, represents how the algorithm might perform if it utilized only an app without the breathalyzer device. Finally, we computed two “base models”: Model 4 used *only* the user’s BrAC estimation for predictions (and no other features), reasoning this represents how accurately users can estimate their own BrAC level without specifically requiring a smart-breathalyzer or an app, and Model 5 represents the “majority class” performance, in which predictions default to the most frequent class. All model parameters are derived from a training set, whereas model performance is evaluated in a separate test set of previously unseen users (python, sklearn, LightGBM). All models were trained with class balancing; hence, we used the macro-averaged *F*1 score.

*AUC* receiver-operating characteristic area under the curve.

### Explainability

**Fig. 3:**
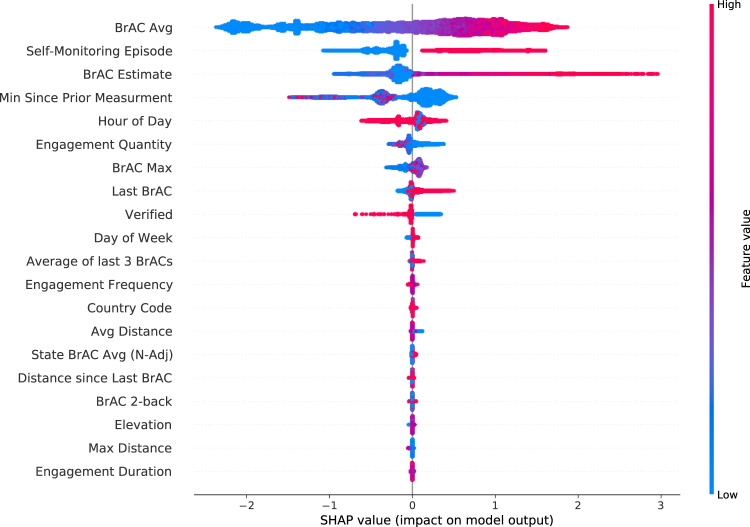
Feature importances. SHAP values quantify the relative impact of each feature on model performance in the hold-out test set of separate users. This figure depicts the most important features, whereby the horizontal location shows whether the feature is associated with a lower (left) versus higher (right) predicted probability of a high BrAC ≥ 0.08 g/dL) across all observations in the test set. The color demonstrates the directionality of the association. These analyses reveal a prominent role for habitual behavior patterns, self-monitoring, engagement, time of day, and human-in-the-loop features such as self-estimations or verifications. They depict secondary roles for other temporal, geographic, policy-relevant, and socioeconomic factors.

Feature importances (quantified herein as gain, or reduction in the loss function per a given feature) can help to inform explainability and intervention design by generating hypotheses regarding the optimal intervention targets. Figure 3 illustrates the ranked Shapley additive explanations (SHAP) feature importance values^16^ in the test set of separate users. The features that contributed the most to model predictions were within-user behavioral factors related to usage or engagement patterns over time, such as measured BrAC trends over time (the average, maximum, and last several recordings), the user’s own subjective BrAC estimate (which is optionally recorded by the user in the app before breathing into the device and receiving the read-out), the number of prior self-monitoring episodes, the quantity and frequency of engagement with the device and app, time since the last BrAC recording, and hour of the day. The remaining features, such as geolocation-based and state- or zip code-level socioeconomic factors, generally had less predictive value in this model.

**Fig. 4:**
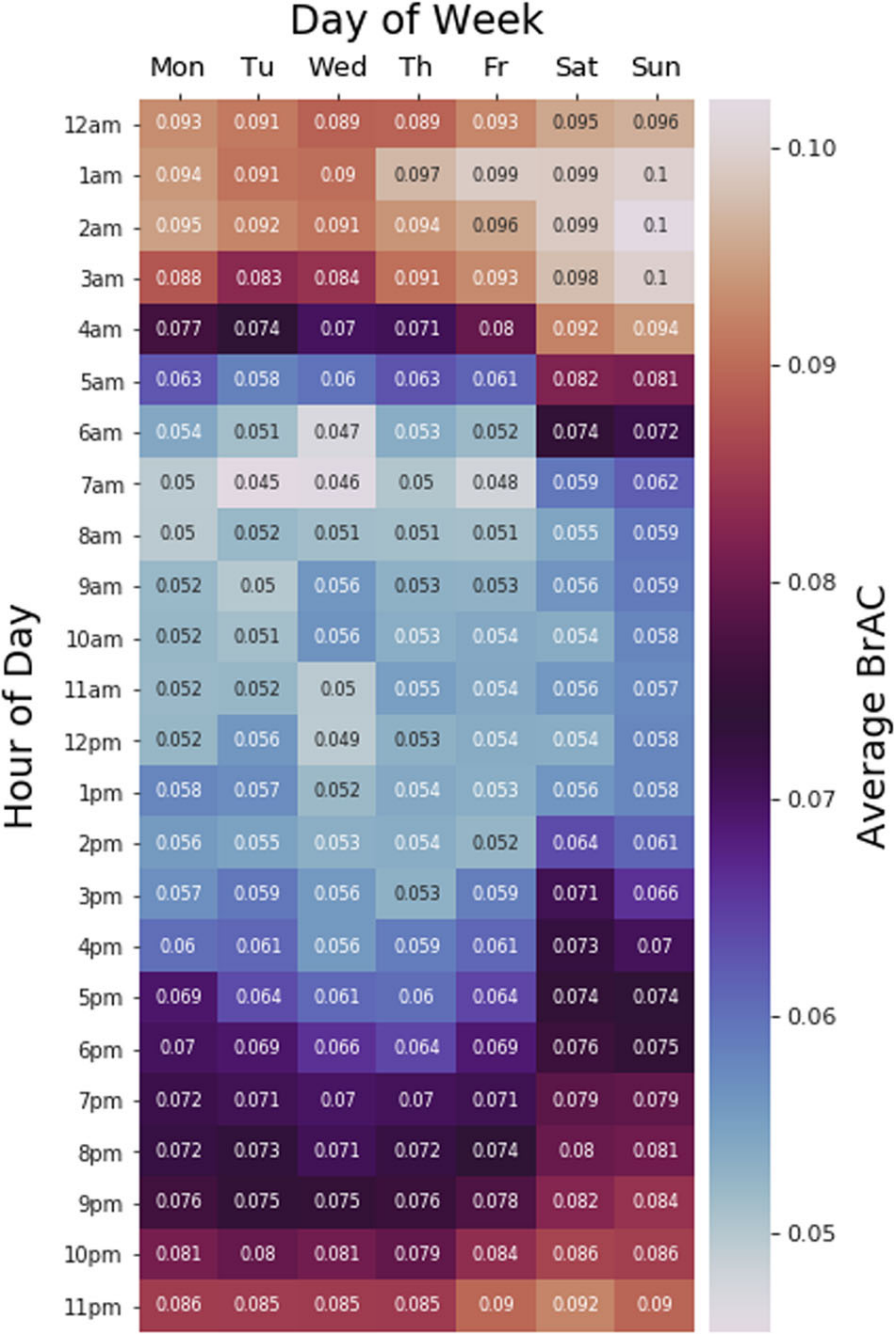
Heat map characterization of BrAC levels by the hour of day and day of the week. The value in each square of the heat map represents the average BrAC level (g/dL) measured across all observations taken during that hour and day of the week, such that white is the highest, red and orange indicate values that exceed the legal limit, and blue values are below the threshold of risk.

Figure 4 displays the average BrAC level for all recordings taken for a given hour on a given day of the week, in order to precisely characterize the temporal patterns in smart-breathalyzer measurements, as a potential predictive feature. BrAC readings are highest between midnight and 2 a.m. on the weekends, and generally in the evenings (note that to provide a more reliable estimate of the BrAC average, we dropped readings in which the user did not verify whether or not they followed the directions to wait 15 min between eating or drinking and taking a BrAC measurement). Supplementary Figure 3 illustrates the number of BrAC observations users provided by day of week and time of day (no observations were excluded), revealing a slight increase in weekday mornings.

**Fig. 5:**
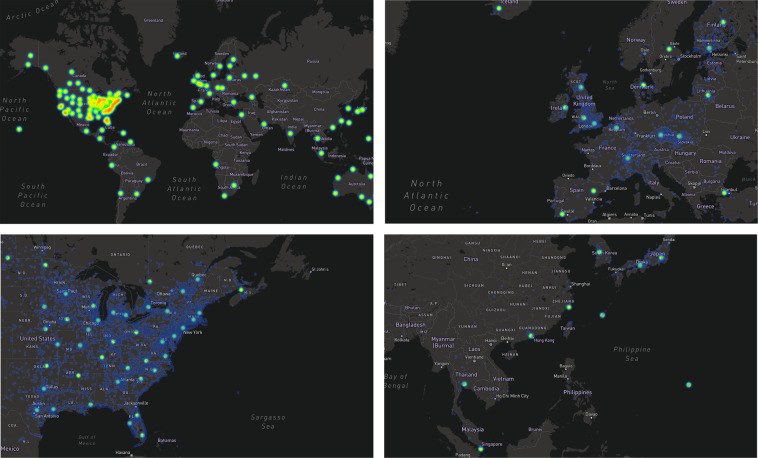
International geospatial characterization of BrAC self-monitoring hot spots using a smart breathalyzer. The dynamic web-based version of this map can be explored by downloading Supplementary Figure 3 and dragging it into a browser window. The green dots represent “hot spots”, or centroids representing clusters of high-density activity, obtained by conducting a K-means analysis on the latitude and longitude of the observations. Blue-purple coloring represents the density (or number) of the actual observations, which become visible upon zooming in further. This map was generated using the Mapbox API (San Francisco, CA) application programming interface, with permission.

Figure 5 (Supplementary Figure 4) displays the geolocations in which BrAC readings were taken. At zoom levels <4, K-means clusters are visualized to identify regions with a high concentration of data. At higher zoom levels, the geolocations of actual BrAC recordings are visualized by a density heat map, but at very high levels, recordings disappear for privacy purposes.

An important target identified by the model was the user’s subjective estimate of their BrAC, considering that self-estimates exhibit substantial inaccuracy^17^, and that interventions to improve insight into one’s BrAC can reduce excess alcohol consumption among moderate drinkers^8^. “BrAC discrimination” was computed as the difference between the subjective BrAC estimate and the objectively measured BrAC level per the smart breathalyzer. The flow of data capture involves: (1) the user being invited to “guess” or estimate their BrAC level in the app before submitting the breath sample, (2) the user breathing into the device, (3) the app provides the actual BrAC read-out, and lastly, (4) the user has the opportunity to enter further notes by navigating deeper into the app. Hence, we studied users’ ability to correctly discriminate their level of intoxication among 443,262 BrAC recordings from 26,646 users. This analysis was limited to those readings in which either the actual or estimated BrAC were above 0, BrAC recordings were verified, and time zone data were available. The median BrAC discrimination value was −0.031 g/dL (−0.061 to −0.009) and the mean was −0.037 ± 0.043 g/dL, indicating that, on average, users’ BrAC estimations were underestimations of their measured BrAC. Mixed modeling analyses revealed that there was a negative correlation between the average within-user BrAC level and BrAC discrimination ability (*B* (SE) = −0.772 (0.005), *t*(d.f. = 26,644) = −143.12, *p* < 0.01 × e^−15^, adj *R*^2^ = 0.435), indicating that, for every 0.01 g/dL increase in a user’s average BrAC, the user tended to underestimate values by an additional 0.008 g/dL. Although subjective BrAC self-estimations *alone* were not highly predictive of objective BrAC values (AUC = 0.64), self-estimates did contribute roughly 3% to model performance (Fig. 2). This contribution is also illustrated in Supplementary Figure 5, which shows that the users underestimate their BrAC most when they are more intoxicated and later at night.

**Fig. 6:**
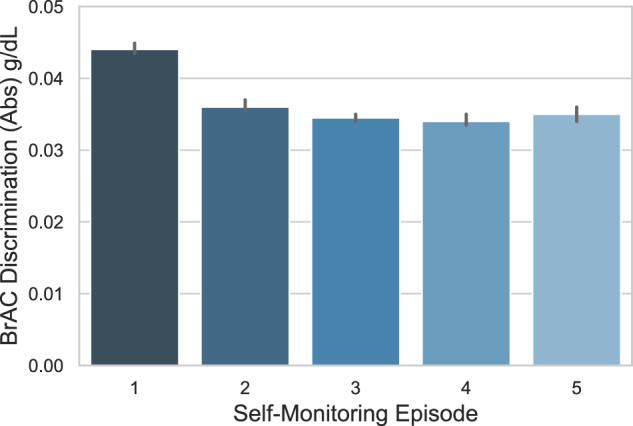
Improvements BrAC discrimination as a function of self-monitoring. Episodes of self-monitoring were defined as any new BrAC measurement occurring at least 12 h after the prior BrAC measurement. Higher values on the *y*-axis denote a greater discrepancy between the true BrAC and the user’s self-estimated BrAC (on average, an underestimate of the true BrAC). Values where both the true BrAC measurement and the user’s BrAC estimate were both zero were excluded, as such values likely indicated product tests or demonstrations rather than true self-monitoring. For this graph, the absolute value of BrAC discrimination values were averaged within each user-episode and graphed as a function of the first five episodes of self-monitoring, with the error bars representing the 95% confidence interval. The number of observations by episode were: (1) 25,503, (2) 19,292, (3) 15,676, (4) 13,193, and (5) 11,593.

The users’ capacity to accurately estimate their BrAC (defined as a lower discrepancy between the estimate and actual value) significantly improved over the first five episodes of self-monitoring (*B* = −0.002, 95% CI: −0.002 to −0.001, *z* = −18.809, *p* < 0.001, 80,833 recordings from 26,056 distinct users; Fig. 6). On average, after the first five episodes of self-monitoring, a user’s estimated BrAC values were <0.01 g/dL closer to the true BrAC value, most of which occurred after the first self-monitoring episode.

## Discussion

Alcohol is one of the leading behavioral causes of global disease burden and mortality^1–3^. In the United States specifically, alcohol mortality rates have doubled over the past two decades^18^. Hence, scalable, low-cost, and easily accessible digital interventions to reduce alcohol-related harms are needed. Breathalyzers or wearable sensors, together with apps and ML algorithms, provide a valuable platform for such interventions. However, to date, no published studies have characterized smart-breathalyzer use and self-monitoring of blood alcohol content under naturalistic conditions. Hence, this study provides a needed foundation for digital intervention development to reduce alcohol-related harms, leveraging a unique dataset of nearly one million recordings in an international sample.

Based on limited information from the smart-breathalyzer and app, this ML model was able to successfully discriminate 85% of recordings with BrAC levels at or above a common legal intoxication threshold (≥0.08 g/dL). To provide a real-world benchmark for this algorithm, it was 21% better at predicting when a user was legally intoxicated than the user’s own subjective estimate, or “best guess”. Moreover, when we removed self-estimated BrAC, model performance remained high (AUC = 82%), relative to the full model (AUC = 85%). In general, we observed graded reductions in model performance when subtracting different feature sets from the full model. Hence, these findings indicate that this model’s predictions represent a complex interaction of individual drinking habits and self-monitoring behavior with environmental, public health contextual factors (e.g., alcohol tax rates, regional urban/rural percentages, and motor vehicle death rates). The most predictive features were various indicators of a prior history of higher heavier drinking, time of day, and the quantity/quality of prior engagement. Geographic features were generally less predictive; nonetheless, one’s geographic region and being closer to the location of one’s most recent BrAC measurement were among the more predictive location-based features. In sum, the final model-derived probabilities may serve as a “digital biomarker” to improve the targeting, monitoring, and personalization of digital health interventions to mitigate alcohol-related harms.

This study demonstrates the feasibility of deriving predictive, ML-based “digital phenotypes of behavior”^12^ from smart-breathalyzer data, in the largest and most internationally diverse user base published to date. Whereas many behavioral studies rely on users’ willingness to answer lengthy surveys and their ability to accurately self-report behavior, smart breathalyzers provide volumes of objective behavioral, temporal, and geographic data. Leveraging these data to perform digital phenotyping has the potential to augment the effectiveness of app-based substance use reduction programs^19^. Such programs are desirable due to the high cost, stigma, and limited access to in-person therapy. Whereas health apps often suffer from poor engagement and high attrition^20,21^, these data revealed a tendency toward longer-term user engagement with the smart breathalyzer.

Many digital health apps rely heavily on the behavioral strategy of self-monitoring^22^, or tracking one’s own behavioral data accompanied by personalized feedback^23^, as a self-regulatory process that is theorized to lead to behavior change. In this study, users underestimated their BrAC by almost 0.04 g/dL on average. Not surprisingly, the more intoxicated a user was, and the later at night it became, the more likely a user was to underestimate his/her BrAC. Prior evidence is consistent with our findings that many people fail to realize when they are legally intoxicated: 37% of bar patrons with BrAC levels >0.08 g/dL reported feeling “no buzz or slightly buzzed”^17^. We found that users’ ability to accurately estimate their BrAC improved slightly over the first few episodes of tracking. However, thereafter, users continued to underestimate their BrAC over longer periods of engagement. Although user-inputted written reflections on the quantity, container sizes, or beverage types might be a useful predictive feature; very few users elected to provide such text notes. In sum, these data indicate that digital self-monitoring *alone* is not enough to fully mitigate alcohol-related risk behaviors. However, an ML algorithm could potentially incorporate users’ subjective BrAC estimates as a “human-in-the-loop” predictive feature, and provide the user with personalized, harm-reduction strategies based on ML-derived insights.

Taken together, the features that emerged as most predictive in this ML model resemble a “digital signature of a habit,” highlighting patterns in BrAC readings, which were defined by their similarity in time, place, and relative to one’s prior behavior. This study engineered autoregressive features representing within-user behavior trends, which provided one means to quantitatively represent alcohol use as a habitual behavior^24^. Furthermore, the desire to drink alcohol can be triggered automatically by cues, which have been repeatedly paired with a rewarding feeling (e.g., alcohol’s euphoric effects), thereby leading to the formation of unconscious associations (cravings). Hence, we reasoned that users of smart breathalyzers may be similarly triggered by temporal and geographically patterned cues—for example, passing a favorite restaurant bar may trigger the desire to drink^25^. This understanding motivated the quantification of the hour of the day from timestamps and distance metrics from geolocation, which both contributed to model predictive accuracy.

Digital biomarkers, similar to this ML model, could inform Precision Behavioral Medicine interventions in several ways^13^. For example, digital biomarkers might track when behaviors are becoming more or less habitual for a given individual (i.e., when prior behavior and temporal/location factors become more or less predictive of future behavior). Such digital biomarkers could then monitor an individual’s response to pharmacologic or behavioral therapies, used to inhibit or desensitize the association between a trigger (cue) and the reward^26–28^. Data visualizations might help users to better identify and recognize their own unique habitual usage patterns and/or triggers. Personalized environments (a familiar bar) evoke stronger reward cue reactivity than standard environments (a generic bar)^29^. Hence, *if* the app were able to access geolocation periodically (as was done in another study to reduce risky drinking^30^), the app could initiate preventative digital coaching when users entered high-risk locations, and *before* users engage with the smart breathalyzer. For example, in anticipation of high-risk times and locations, the app might nudge a user to engage a healthier substitution behavior or self-care strategy, thereby preempting cravings.

In terms of validity, a prior study reported that BrAC measurements by this device were as highly correlated with actual blood alcohol levels as a police-grade device^6,7^. We recognize that BrAC levels can be spuriously inflated by mouth fumes if users neglect the app instructions to wait at least 15 min between consuming alcohol and taking a measurement. However, the app feature enabling users to verify a recording appears to help identify this user behavior as a source of measurement error; therefore, our ML model included it as a predictive factor. Specifically, the coloring and directionality of the SHAP values for this user-verification feature (Fig. 3) indicate that verification was primarily used by the model to improve predictions of low BrAC, whereas the absence of verification was a weaker predictor of high BrAC. It is possible that the behavior of failing to validate might be a proxy marker for impulsivity or poorer attentional control, and thus constitutes valuable information to include.

We found a significant association between the state’s average BrAC level and the motor vehicle death rate, which supports the notion that smartphone-enabled devices provide data that may usefully inform public health and policy interventions. Although speculative, this initial evidence supports the validity of naturalistic BrAC measurements as a new index of naturalistic consumption, relevant for studying public health or epidemiologic questions. Moreover, the Colorado Department of Transportation recently announced a public health initiative to provide smart breathalyzers (from BACtrack) to individuals who recently received a first DUI in advance of Labor Day, to help prevent these individuals from getting a second DUI^31^. Hence, future studies could attempt to verify whether temporal patterns in BrAC measurements are responsive to population-scale interventions utilizing smart breathalyzers.

### Limitations

This study has several limitations. Although we excluded recordings in which users reported that they had lent their device to another person, and where needed, we excluded or adjusted for unverified recordings, it remains likely that some measurement noise was introduced by unreported user error. Predictions may also be slightly less accurate for users whose location services are turned off, although geographic features were not among the most predictive. For future predictive models, it will be important to focus on models that anticipate drinking onset; however, that would likely require the device to gather more extensive data in the background when the user is not in the app, which was not available in this study. Moreover, users and regulatory bodies both are increasingly wary of data privacy concerns. Nonetheless, interventions at the onset of a drinking episode can still be clinically relevant from a harm-reduction standpoint^4,32^. Finally, we do not know the sampling biases inherent in which individuals choose to purchase or use a smart breathalyzer; hence, such biases could conceivably reduce the generalization of the findings. However, the large size and geolocation-based diversity of the user base, combined with observed correlations with US population-level health data for alcohol-associated motor vehicle death rates, implies that the results have sufficient generalizability to be relevant to larger-scale health concerns.

In conclusion, alcohol use is a major preventable behavioral cause of morbidity and mortality, and the application of ML algorithms to smart-breathalyzer data has the potential to drive innovative, large-scale, cost-effective preventative interventions. These results provide the first large-scale, global “snapshot” of naturalistic usage patterns for smart-breathalyzer devices. We provide the proof of concept that risk-associated BrAC levels can be predicted with high accuracy simply from the “digital exhaust” of the user’s interaction with a smart breathalyzer and associated app. Hence, this characterization informs future hypothesis generation for Just-in-Time Precision Medicine interventions guided by ML.

## Methods

### Participants

We analyzed 973,264 unique BrAC observations from 33,452 distinct users, collected using a commercially available smart breathalyzer (BACtrack, San Francisco, CA) between May of 2013 and June of 2017. Data were provided to researchers at the University of California, San Francisco through a data-sharing agreement. Studies validating certain BACtrack breathalyzer models against police-grade devices report similar accuracy^6,7^. The BACtrack App syncs with the Bluetooth-enabled breathalyzer to collect data, only from users who have their data storage activated and location services turned on. Users were prompted to enter their “best guess” (estimate) of their BrAC before exhaling into the device. Demographic data such as gender, age, or race/ethnicity were not available, and device ids were not included in the dataset for deidentification purposes. In addition to BrAC measurements (reflecting momentary estimates of users’ BAC), the initial dataset consisted of timestamps, geolocation, and a limited number of other engagement features, including users’ BrAC estimates and verification that they followed app instructions to wait 15 min from the last sip prior to taking a BrAC measurement (verified). Users could indicate whether a given BrAC measurement was provided by themselves or another individual (user-provided). We removed all observations (14.9%) that were reported as not user-provided, and any duplicate observations, but we retained unverified measurements (~12%) in order to quantify their potential impact. Users also had the option to provide a drink count, profile photos, and write free-form text notes, although these data were sparse. The company BACtrack was not involved in the design or funding of this study. The University of California, San Francisco Institutional Review Board approved this study, including waiver of consent to utilize these deidentified data.

The commercial smart-breathalyzer company BACtrack (San Francisco) provided researchers at the University of California, San Francisco, Division of Cardiology, School of Medicine, with a deidentified dataset including 1,268,329 total rows of data, collected between May of 2013 and June of 2017. After rigorous data cleaning, the final study included 973,264 total BrAC measurements, recorded by 33,452 distinct users. To clean the data, we excluded 146,244 values (14.9%) that users indicated did not belong to them (e.g., in the app, users can indicate that they lent the device to a friend), as they would likely reduce model predictive capability. In addition, data were excluded per the following criteria: (1) 30,056 duplicate rows, (2) 102,531 rows missing a user id, and (3) 1350 rows with technical errors in the translation of the GMT timestamp to local time. An additional five rows were excluded due to BAC levels exceeding 0.50 as likely technical errors or fatal^1^. Although very high BAC levels (e.g., those >0.25) might potentially be spurious, they might also conceivably be biologically plausible; hence, we utilized a nonparametric algorithm that can manage potential outliers. Of the remaining rows, 120,989 BrAC observations were not “verified”, indicating that participants may not have followed the instructions not to eat or drink during the 15 min period prior to BrAC measurement. We retained these observations in the analyses because a practically useful algorithm must be robust to expected deviations from “ideal” user behavior, and we were confident that the tree algorithm would exhibit robust predictive performance so long as verification was included as a feature in the model.

### External validity investigation

To assess whether BrAC recordings relate to regional variation in alcohol-related harms, we tested whether higher BrAC levels, averaged by state, would correlate with death rates from alcohol-related driving. We downloaded data from the Centers for Disease Control and Prevention (CDC) on state-level rates of death from impaired driving (per 100,000 population) for people killed in crashes involving a driver with a BAC ≥ 0.08 g/dL for the year 2014^33^. State average BrAC values were based on 53,674 BrAC observations from 2641 distinct users from 50 states in 2014, and were normalized to the square root of the number of users for that state. The association between motor vehicle death rates and BrAC levels, by state, were analyzed using robust linear regression models in Python 3 (statsmodels).

### Self-monitoring and engagement

To represent the digital signature of alcohol consumption, myriad statistical features were constructed using time-series of BrAC measures. For each BrAC observation used as the label to supervise the ML algorithm, BrAC features were constructed based only on the time-series of BrAC levels previously measured for that user, thereby permitting indices of prior behavior to predict future behavior. Features included the average, minimum, maximum, range, median, quartiles, and the interquartile range of all prior BrAC observations previously recorded for that user. As recent behaviors may be stronger predictors than older behavior, we also created a set of lagged variables, using the prior three BrAC observations.

The breathalyzer app allows users to self-report a BrAC “guess”, or estimate, during the time between activating the device and recording the breath sample. Evidence shows that helping the user recognize the gap between a user’s perceptions and his/her actual objective alcohol measurement is a potentially useful component of alcohol behavior change interventions^8^. Hence, we quantified “BrAC discrimination” as the discrepancy between a user’s subjective estimate of their BrAC and their actual BrAC level, such that a negative number represents an underestimate of the true BrAC. We sought to test whether or not a user’s subjective BrAC estimates became more accurate, the more episodes they self-monitored.

The *duration* of engagement with the device was quantified as the number of days of engagement from the first to the current BrAC observation. To assess the *quantity* of engagement, we computed the number of BrACs each user previously measured. To assess the *frequency* of engagement, we assessed the number of days on which users measured at least one BAC; for example, this metric differentiates users who measured BrAC multiple times in a day versus those who measured once a day for 10 days. Because some users measured their BrAC often within a short period (e.g., 15 min), we also quantified frequency by “episodes” of self-monitoring. We quantified a new self-monitoring episode as a new BrAC recording occurring ≥12 h after that same user’s prior recording.

Several less common forms of deeper engagement with the app were additionally quantified. The presence of a photo logged by a user with the app was included as a feature (10,931 or 1.14% of the observations included a photo; the actual photos themselves were not included in the dataset). Second, users had the option of logging the number of drinks consumed, which occurred in 8415 observations (0.87%).

Users could enter a note as a free-text field in the app, although the prevalence was sparse with 930 notes (0.1% of observations). We used natural language processing (NLP) to investigate *why* users self-monitor, *what* they focus on when they self-monitor, and *how* such reflections are associated with BrAC levels. NLP features were quantified using regular expressions to detect language reflecting the numeric amount, container type, and beverage type, as well as the presence/absence of a note. A feature for the binary existence of a note (“has note”: yes/no) was created. In addition, we sought to quantify features for a model prediction that reflect user self-monitoring of the amount, type, and impact of alcohol consumption. We hypothesized that greater user engagement with the app, in terms of entering cognitive reflections to quantify or characterize their alcohol consumption would help predict a lower BrAC, although we cannot strictly test that in this cross-sectional design. Regular expressions in Amazon Web Services Redshift’s SQL language were used to extract features representing the count of instances per note of the following: (1) numeric amounts, (2) volumetric measurement amounts (e.g., “glass, bottle, tumbler”), and (3) alcohol content (e.g., beer, wine, and whiskey). A final NLP self-monitoring feature was derived as the sum of these counts.

### Temporal features

Minutes since a given user’s last BrAC measurement were computed as a feature for analysis, based on Universal Time Coordinated (UTC) timestamps. To compute hour of day and day of week as additional features, user local timestamps were first quantified, based on time zones and UTC timestamps, where data were available. Dates considered holidays were integrated, given that drinking may be more prevalent on holidays. Because the majority of the users were based in the United States, and more drinking occurs in conjunction with certain sporting events, we also created separate features representing dates of the Superbowl, World Series, and NBA Championships, and whether a given user’s observation was taken in a winning or losing state.

### Location-based features

For users living in the United States, zip codes were provided with the dataset, which corresponded to where the BrAC measurement was taken. In order to quantify whether a given zip code was predominantly urban or rural, we merged data from the United States Census Bureau^34^ regarding core-based statistical areas and their relationship to zip codes^35^. Features were created to reflect the estimated population size of each zip code, and the percentage of each zip code defined as an urban or a rural area (<50,000 individuals per the US Census definition)^36^. Zip codes were not entered directly into the GBCT model to avoid the need for very deep trees, which would have promoted model overfitting. However, the total count of the zip codes, states, and countries in which each user had ever self-monitored BAC levels was added as features.

The dataset provided analyzable geolocation data for 66% of BAC observations. As raw geolocation data cannot be effectively inputted and analyzed by the GBCT model, K-means clusters were computed to designate regions with a high density of data and distance features were engineered from the geolocation data as described below. Distances between measurements may be useful to indicate habitual drinking spots, variability in drinking venues, or “jet-setter” lifestyles. We used the following formula to calculate distances between subsequent geolocations in Redshift (see Supplementary information; https://github.com/kaschbacher/bac). To compute distances, we computed the distance between the locations where a user measured his or her BAC at time “*t*” versus his/her previous BAC (time “*t* − 1”). We then took the distribution of distances within each user and computed additional statistical features to enter into the GBCT model: the average distance, min and max distance, median and quartiles of distance, the interquartile range, and the coefficient of dispersion, which can help identify users who tended to measure the majority of their BACs in one location, with few trips over longer distances. Elevation was provided in the initial dataset and was included as a feature.

BACtrack users are given the option to verify whether they followed the instructions to wait a minimum of 15 min between drinking a beverage or eating something and taking a measurement, which, if not followed, may artificially inflate a BrAC reading. However, as this feature is small and toward the bottom of the screen, some users who have unverified readings may not have seen it. We conducted mixed modeling analyses (Python, statsmodels) to better understand whether the validity of BrAC readings was likely adversely affected by users who did not follow the instructions to wait a sufficient time between drinking and taking a reading, that is, “unverified” readings.

As taxes tend to beneficially impact alcohol-related health risks at the population level^37^, we incorporated state-level data from 2014 (the latest available and best overlap with BrAC year) on state taxes placed on alcohol, as well as sales, gas, and cigarette tax rates^38^.

As alcohol use is correlated with income and structural features of the environment^39^, we merged information from the US Census Bureau quantifying the official estimated number of individuals living in poverty, in units of thousands, per state^40^.

We integrated information on the prevalence of heavy drinking per state from the Center for Disease Control on chronic disease indicators, as a means to bridge between digital health user-level data and epidemiologic factors^41^.

### Data analyses

Descriptive statistics and simple statistical feature engineering were run using PostgreSQL queries, using counts and percentages, means and standard errors, medians and the interquartile range, or lagged computations, as appropriate.

A robust linear regression analysis (Python, statsmodels) was conducted to determine whether higher state average BrAC levels were significantly associated with higher motor vehicle death rates, adjusted for the square root of the number of users per state. In addition, we conducted mixed modeling analyses (Python, statsmodels) to determine whether certain user behaviors (not verifying their reading, testing the device) were associated with BrAC levels.

We trained a stochastic GBCT model using the LightGBM algorithm in Python 3 (scikit-learn API). Data were randomly split into training (70%), validation (10%), and test sets (20%), which did not contain overlapping users. We fit model parameters in the training set, and tuned hyperparameters in the validation set. Based on a validation set, with a default learning rate (0.1), early stopping (*n* = 5), and moderate regularization (L1 = 0.5, L2 = 0.5), the optimal number of trees was determined to be 89. We conducted a model evaluation in an independent test set, with no overlapping users, using the receiver-operating characteristic area under the curve as the primary metric, with sensitivity (recall), specificity, and precision as secondary metrics, using a threshold of 0.5. We filled missing data with a value of −999, which has the advantages that: (1) all data were included in the analysis (no biases are introduced by the exclusion of incomplete data observations), and (2) the tree algorithm then used “missingness” as a predictive feature in the algorithm by splitting on low negative values.

To conduct feature selection, we conducted correlation analyses across 98 features, and highly correlated sets of features were either combined statistically or a single a priori indicator was selected, yielding 46 features. To identify the features with the greatest impact on predictions, feature importances were computed using SHAP values^16^. The final model was evaluated in the separate test set using the constrained feature set and hyperparameter settings derived from the validation set.

In order to facilitate future intervention development, we conducted additional statistical analyses to better interpret features with high importance, which might play a central role in the delivery of a precision medicine intervention. Visualizations of temporal and geographic patterning of BrAC levels were generated a via Python (open source), R (open source), plotly (Montreal, Canada), Highcharts (Vik i Sogn, Norway), and MapBox API (San Francisco, CA). To visualize the primary geographic clusters of BrAC observations, a K-means analysis (Python, sklearn.cluster.KMeans) with 120 clusters was run on the latitude and longitude of all observations.

To test whether individuals who use the device more frequently show improvements in BrAC discrimination (accuracy in estimating BrAC), we conducted a mixed-model linear regression analysis (python, statsmodels) using self-monitoring episode (level 1) within user (level 2) as a predictor of BrAC discrimination. We limited the analyses to observations in which either the actual or estimated BrAC value was not zero, and data for the BrAC estimate and local timestamp were available. Because we anticipated that improvement due to self-monitoring would be most pronounced during the initial period of use, we further limited the data to the first five episodes. To improve the normality of the data and eliminate statistical outliers in our outcome variable (BrAC discrimination), we excluded datapoints <2.5% of the distribution or >97.5% of the distribution. Data were aggregated to obtain the mean absolute value of the BrAC discrimination value for each user in each episode; hence, a decline over subsequent episodes of self-monitoring would indicate that users’ accuracy in estimating their BrAC improved with subsequent episodes.

### Reporting summary

Further information on research design is available in the Nature Research Reporting Summary linked to this article.

## Supplementary information

Supplementary Information

Reporting Summary

## Data Availability

The datasets generated during and/or analyzed during the current study are available from the corresponding author on reasonable request, but may require data-sharing agreements with the private company (BACtrack, San Francisco, CA) that originally collected the measurements from their mobile application.

## Code availability

The ML code is provided in github (https://github.com/kaschbacher/bac). Visualizations of temporal and geographic patterning of BrAC levels were generated via Python (open source), R (open source), plotly (Montreal, Canada), Highcharts (Vik i Sogn, Norway), and MapBox API (San Francisco, CA). To visualize the primary geographic clusters of BrAC observations, a K-means analysis was used (Python, sklearn.cluster.KMeans). Statistical analyses were performed using Python (statsmodels.api and sklearn) for general linear models or Redshift Postgres SQL for descriptives. Computations of distances between subsequent geolocation points were performed in Redshift Postgres SQL. AWS S3, Redshift Postgres SQL, and Spectrum were used to build the database. Further details on variables and parameters can be found in the methods descriptions, the Supplementary information, in the github codebase referenced herein.
