# Supplementary material for: Machine learning prediction of blood alcohol concentration: a digital signature of smart-breathalyzer behavior
Source: NPJ Digit Med. 2021 Apr 20;4:74. doi: 10.1038/s41746-021-00441-4 (PMC8058037; doi:10.1038/s41746-021-00441-4)
Supplement: Supplementary file 1 — Supplementary Information [file 41746_2021_441_MOESM1_ESM.pdf]

Supplementary Information

Results

Sources of User Error: Quantification of Potential Effects on BrAC readings

We examined potential issues related to the verification of BrAC readings in subset of 443,262 BrAC readings from 26,646 users, who we deemed unlikely to be testing or demonstrating the device because either the observed BrAC reading or the BrAC estimation were above zero. Linear mixed model analyses revealed that, as expected, BrAC readings that users did not verify (M±SD: 0.091g% ± 0.077, n=76,753) were significantly higher than verified BrAC readings (M±SD: 0.078g% ± 0.068, n=446,540) (B=-.017, 95% CI: -.017 – -.016, z= -52.623, p<.001). Where possible, we included verified as a statistical covariate to adjust for this factor rather than discard the observations. The GBCT algorithm can handle verification as a feature that may interact with other features to adjust the predictions. Moreover, verification is a form of user behavior that might usefully reflect cognitive tendencies toward impulsivity, which contribute to behavioral risk for addictive or reward-driven behaviors;[2] hence, we suggest that merely removing the observations might introduce a sampling bias and reduce the generalizability of the machine learning predictions.

References

1. Gigli, F., Lagioia, G., Morini, O., Paoletti, R. & Sironi, L. High Blood Alcohol Levels in Cadaverous Blood. in *Acta Medicinæ Legalis Vol. XLIV 1994* 417–420 (Springer Berlin Heidelberg, 1995). doi:10.1007/978-3-642-79523-7_133

2. Coskunpinar, A., Dir, A. L. & Cyders, M. A. Multidimensionality in Impulsivity and Alcohol Use: A Meta-Analysis Using the UPPS Model of Impulsivity. *Alcohol. Clin. Exp. Res.* **37**, 1441–1450 (2013).

Supplementary Figure 1:
The Number of BrAC Recordings as a Function of Month-Year and User-Cohorts

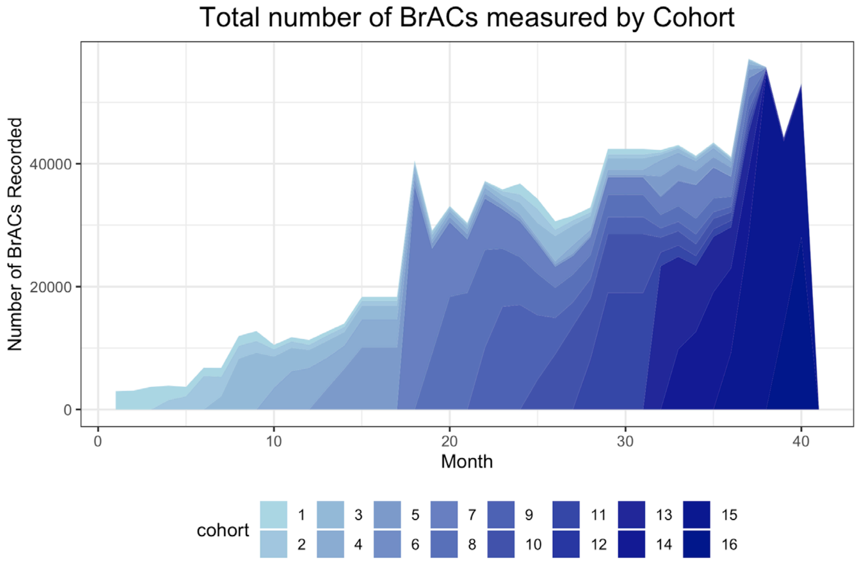

Note: The figure displays a cohort analysis of the number of BrAC observations gathered in each month over the period of data collection. Each user was assigned to a cohort based on the year and month in which they provided their first BrAC observation. Each cohort encompassed a 3-month window of time. For any given cohort, the height of that group's area corresponds to a metric of how engaged that cohort of users was during that month. As new app versions, devices and products, technical issues, and company sales initiatives can all impact the quantity of recordings at any point in time, cohort analyses assist in tracking how such factors may combine with historical or seasonal trends to impact user engagement and available data. Data from months 16-17 and 30-31 (as depicted above) were incomplete as provided; hence, for the purposes of effective visualization, the data from months 15 and 29 were carried forward to the subsequent two incomplete months.

Supplementary Figure 2:

Heat Map Characterization of BrAC Self-Monitoring Behavior by Hour and Day of Week

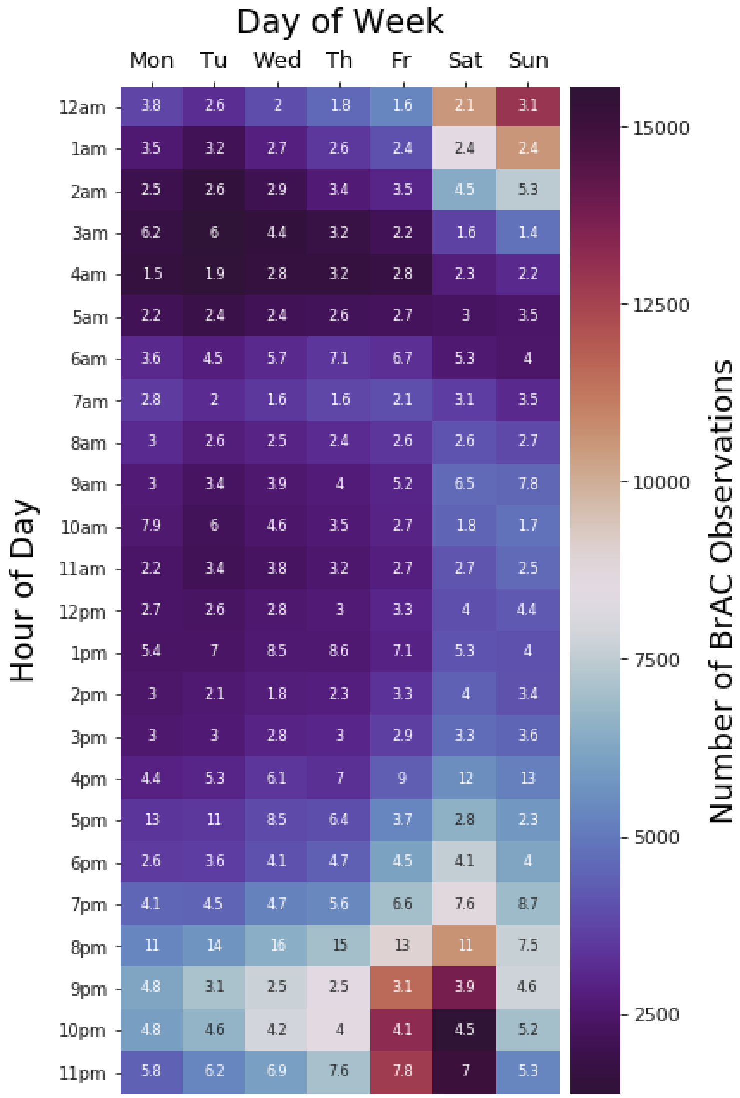

Note: The value in each square of the heat map represents the number of BrAC observations made, per 1000 observations, during each hour and day of week. Square that are orange/red indicate times when many users were self-monitoring, whereas dark blue represent the times in which the least self-monitoring was occurring.

Supplementary Figures 3, 4, and 5 are dynamic files and therefore are accessed via the links below:

Supplemental Figure 3

https://greg-marcus-paper-blood-alcohol.s3-us-west-2.amazonaws.com/Sup-Figure-3-State-Map-BrAC-MVD-2021-01-17.html

Supplemental Figure 4

https://greg-marcus-paper-blood-alcohol.s3-us-west-2.amazonaws.com/Supp-Figure-4-MapboxAPI-2019-07-08.html

Supplemental Figure 5

https://greg-marcus-paper-blood-alcohol.s3-us-west-2.amazonaws.com/Supp-Figure-5-Scatter-level-vs-guess-2019-08-02.html

Supplementary Table 1: The Number of Distinct Users and Observations by Country

Country Code	Country	Number of Users	Number of Observations
AD	Andorra	1	1
AR	Argentina	17	136
AT	Austria	33	252
AU	Australia	1275	24698
AX	Åland Islands	4	6
BE	Belgium	53	1012
BG	Bulgaria	12	127
BR	Brazil	94	742
CA	Canada	2165	38965
CH	Switzerland	701	9909
CZ	Czechia	282	4004
DE	Germany	129	1354
DK	Denmark	25	535
DO	Dominican Republic	22	215
ES	Spain	67	628
FI	Finland	306	5320
FO	Faroe Islands	2	4
FR	France	162	1739
GB	United Kingdom of Great Britain and Northern Ireland	668	12518
GT	Guatemala	8	59
GU	Guam	8	44
HR	Croatia	6	40
HU	Hungary	15	68
IM	Isle of Man	1	1
IN	India	53	391
IS	Iceland	23	254
IT	Italy	109	725
JE	Jersey	1	4
JP	Japan	168	6930
LI	Liechtenstein	1	12
LK	Sri Lanka	5	31
LT	Lithuania	63	1454
LU	Luxembourg	5	52
MC	Monaco	3	10
MD	Republic of Moldova	1	1
MX	Mexico	142	1088
MY	Malaysia	40	486
NL	Netherlands	37	428
NO	Norway	39	410
NZ	New Zealand	151	1734

PH	Philippines	8	28
PK	Pakistan	3	14
PL	Poland	113	1269
PR	Puerto Rico	24	112
PT	Portugal	23	243
RO	Romania	9	63
RU	Russian Federation	197	4716
SE	Sweden	33	413
SI	Slovenia	3	20
SK	Slovakia	75	755
TH	Thailand	43	561
TR	Turkey	46	730
US	United States of America	21115	503112
VI	United States Virgin Islands	9	61
XX	Unknown	16404	344313
ZA	South Africa	39	467

Supplementary Table 2: The Number of Distinct Users and Observations by United States

State	Number of Users	Number of Observations
Alabama	180	1836
Alaska	146	2311
Arizona	758	12579
Arkansas	107	1238
California	4616	71485
Colorado	1299	28087
Connecticut	329	5659
Delaware	100	1749
District of Columbia	152	1144
Florida	1428	23231
Georgia	550	8587
Hawaii	183	2597
Idaho	161	5155
Illinois	1026	18429
Indiana	553	8897
Iowa	299	4383
Kansas	258	4879
Kentucky	287	7132
Louisiana	283	3106
Maine	115	1211
Maryland	574	9298
Massachusetts	629	11270
Michigan	948	22345
Minnesota	841	17357
Mississippi	100	1613
Missouri	604	8123
Montana	97	1134
Nebraska	237	4485
Nevada	641	5212
New Hampshire	184	4687
New Jersey	661	8853
New Mexico	122	2018
New York	1266	19045
North Carolina	806	16122
North Dakota	138	1681
Ohio	612	9768
Oklahoma	213	5062
Oregon	514	10213
Pennsylvania	924	13209
Rhode Island	113	1522
South Carolina	338	4631
South Dakota	117	2312

Tennessee	402	5334
Texas	1816	42906
Utah	264	4096
Vermont	126	1517
Virginia	845	13294
Washington	1114	18437
West Virginia	129	1549
Wisconsin	602	10448
Wyoming	69	1399
(missing)	1441	10477

Supplementary Table 3: The Number of Distinct Users and Observations by Year

Year	Number (%) of Users	Number (%) of Observations
2013	1817 (5.43)	42847 (4.4)
2014	3453 (10.32)	94407 (9.7)
2015	13579 (40.59)	414619 (42.6)
2016	11929 (35.66)	268218 (27.56)
2017	9609 (28.72)	153173 (15.74)

Note: These data do not represent the complete count of all BACtrack users. Data for 2013 began on May 1st and went through June 11th of 2017. Product marketing campaigns and other macroeconomic factors may also impact the number of users or observations taken in any given year.

Supplementary Table 4: Machine Learning Model Feature Descriptions

Feature	Category	Data Source	Feature Description
BrAC Estimate	behavioral	BACtrack app	User-reported BrAC estimate, entered in the app during the time between activating the device and receiving the measured BrAC output
BrAC Avg	behavioral	BACtrack smart-breathalyzer	Average BrAC of user's prior measurements
Self-Monitoring Episode	behavioral	BACtrack smart-breathalyzer	New self-monitoring episodes were quantified as a new BrAC measurement occurring ,>=12 hours after the prior recording
Min Since Prior Measurement	temporal	BACtrack smart-breathalyzer	Minutes since prior BrAC measurement
Hour of Day	temporal	BACtrack smart-breathalyzer	Hour of day during which BrAC measurement was taken (local time)
Verified	behavioral	BACtrack app	User-reported verification that he/she followed the instructions to obtain a maximally accurate BrAC measurement
BrAC Max	behavioral	BACtrack smart-breathalyzer	Maximum BrAC of user's prior measurements
App Engagement (note, photo, drink counts)	engagement	BACtrack app	App Engagement (recording notes, photos, or drink counts)
Engagement Quantity	engagement	BACtrack smart-breathalyzer	The number of prior BrAC measurements for a given user
Last BrAC	behavioral	BACtrack smart-breathalyzer	User's last BrAC measurement
BrAC 2-back	behavioral	BACtrack smart-breathalyzer	User's second to last BrAC measurement
Last 3 BrAC	behavioral	BACtrack smart-breathalyzer	The average of the user's last 3 BrAC measurements
BrAC Min	behavioral	BACtrack smart-breathalyzer	Minimum BrAC of user's prior measurements
Country Code	geographic	BACtrack app	Country
Year	temporal	BACtrack smart-breathalyzer	Year of BrAC measurement
Max Distance	geographic	BACtrack app	Maximum distance (km) travelled between subsequent BrAC measurements (geolocation-based)
State BrAC	geographic	BACtrack smart-	Average BrAC level by state

Avg		breathalyzer	
Engagement Duration	engagement	BACtrack smart-breathalyzer	Number of days between a user's first and last BrAC measurement
Day of Week	temporal	BACtrack smart-breathalyzer	Day of Week (categorical)
Elevation	geographic	BACtrack app	Elevation (geolocation-based
Engagement Frequency	engagement	BACtrack smart-breathalyzer	The number of days on which a user measured his/her BrAC
Avg Heavy Drinking Male	health	Center for Disease Control, chronic disease indicators	Prevalence of heavy drinking per state for males
State Wine Tax/Gal	socioeconomic	Tax Foundation	State taxes on wine
State Beer Tax/Gal	socioeconomic	Tax Foundation	State taxes placed on beer
Avg Distance	geographic	BACtrack app	Average distance (km) travelled between subsequent BrAC measurements (geolocation-based)
Avg Heavy Drinking	health	Center for Disease Control, chronic disease indicators	Prevalence of heavy drinking per state
State Spirit Tax/Gal	socioeconomic	Tax Foundation	State taxes placed on spirits
State BrAC Avg (N-Adj)	geographic	BACtrack smart-breathalyzer	State BrAC average, adjusted for the number of observations per state
Impaired Driving MVD	health	Centers for Disease Control and Prevention (CDC)	State-level impaired-driving death rates (per 100,000 population) for people killed in crashes involving a driver with a BrAC,â•0.08 g/dL
% in Poverty/State	socioeconomic	United States Census Bureau	The official estimated percentage of individuals living in poverty, in units of thousands, per state
No. prior BrACs	behavioral	United States Census Bureau	The difference between the official estimated percentage of individuals living in poverty per state and the supplemental figures, which integrate additional home equity and childcare information
State Code	geographic	BACtrack app	US state
No. Users per State	geographic	BACtrack smart-breathalyzer	The number of distinct device users per state
Avg Heavy Drinking Female	health	Center for Disease Control, chronic disease indicators	Prevalence of heavy drinking per state for females
% of Zip Urban	geographic	United States Census Bureau	The percentage of each zip code defined as living in an urban area

Game	temporal	BACtrack smart-breathalyzer	Dates of the Superbowl, World Series and NBA Championship games
State Sales Tax/Gal	socioeconomic	Tax Foundation	State sales taxes rates
State of Game	geographic	User geolocation/Manual Entry of Games	US State where game was played
Distance since Last BrAC	geographic	BACtrack app	Distance (km) travelled since the last BrAC measurement (geolocation-based)
Month of Year	temporal	BACtrack smart-breathalyzer	Month of the year
Holiday	temporal	BACtrack smart-breathalyzer	Dates of major bank holidays
Distance/Hour	geographic	BACtrack app	Distance (km)/hours since the last BrAC measurement (geolocation/timestamp-based)

Note: These features were selected from a larger group of engineered features based on inspection and minimization of feature collinearity, *a priori* knowledge derived from the scientific literature, neuroscience and behavioral literature on habit formation and addiction, and data science experience in time series and mixed modeling analyses. Please also see Figure 4 in the main manuscript for Shapley values of the most important features.
