# Supplementary material for: Machine learning prediction of blood alcohol concentration: a digital signature of smart-breathalyzer behavior
Source: NPJ Digit Med. 2021 Apr 20;4:74. doi: 10.1038/s41746-021-00441-4 (PMC8058037; doi:10.1038/s41746-021-00441-4)
Supplement: Supplementary file 2 — Reporting Summary [file 41746_2021_441_MOESM2_ESM.pdf]

nature research

Corresponding author(s): *Double-blind peer review submissions: write DBPR and your manuscript number here instead of author names.*

Last updated by author(s): *YYYY-MM-DD*

Reporting Summary

Nature Research wishes to improve the reproducibility of the work that we publish. This form provides structure for consistency and transparency in reporting. For further information on Nature Research policies, see our Editorial Policies and the Editorial Policy Checklist.

Statistics

For all statistical analyses, confirm that the following items are present in the figure legend, table legend, main text, or Methods section.

n/a	Confirmed	
☐	☒	The exact sample size (*n*) for each experimental group/condition, given as a discrete number and unit of measurement
☐	☒	A statement on whether measurements were taken from distinct samples or whether the same sample was measured repeatedly
☐	☒	The statistical test(s) used AND whether they are one- or two-sided<br>*Only common tests should be described solely by name; describe more complex techniques in the Methods section.*
☐	☒	A description of all covariates tested
☐	☒	A description of any assumptions or corrections, such as tests of normality and adjustment for multiple comparisons
☐	☒	A full description of the statistical parameters including central tendency (e.g. means) or other basic estimates (e.g. regression coefficient) AND variation (e.g. standard deviation) or associated estimates of uncertainty (e.g. confidence intervals)
☐	☒	For null hypothesis testing, the test statistic (e.g. F, t, r) with confidence intervals, effect sizes, degrees of freedom and P value noted<br>*Give P values as exact values whenever suitable.*
☒	☐	For Bayesian analysis, information on the choice of priors and Markov chain Monte Carlo settings
☐	☒	For hierarchical and complex designs, identification of the appropriate level for tests and full reporting of outcomes
☐	☒	Estimates of effect sizes (e.g. Cohen's *d*, Pearson's *r*), indicating how they were calculated

Our web collection on statistics for biologists contains articles on many of the points above.

Software and code

Policy information about availability of computer code

Data collection	Data was collected from BACtrack Bluetooth-enabled breathalyzers (provided by BACtrack, San Francisco, CA); We downloaded data from the Centers for Disease Control and Prevention (CDC) on state-level rates of death from impaired driving (per 100,000 population) for people killed in crashes involving a driver with a BAC ≥ 0.08 g/dL for the year 2014
Data analysis	Python 3 (statsmodels) to customize a machine learning code. . The machine learning code is provided in github (https://github.com/kaschbacher/bac).

For manuscripts utilizing custom algorithms or software that are central to the research but not yet described in published literature, software must be made available to editors and reviewers. We strongly encourage code deposition in a community repository (e.g. GitHub). See the Nature Research guidelines for submitting code & software for further information.

Data

Policy information about availability of data

All manuscripts must include a data availability statement. This statement should provide the following information, where applicable:

- Accession codes, unique identifiers, or web links for publicly available datasets
- A list of figures that have associated raw data
- A description of any restrictions on data availability

The datasets generated during and/or analysed during the current study are available from the corresponding author on reasonable request.

Field-specific reporting

Please select the one below that is the best fit for your research. If you are not sure, read the appropriate sections before making your selection.

☒ Life sciences ☐ Behavioural & social sciences ☐ Ecological, evolutionary & environmental sciences

For a reference copy of the document with all sections, see nature.com/documents/nr-reporting-summary-flat.pdf

Life sciences study design

All studies must disclose on these points even when the disclosure is negative.

Sample size	973,264 user-initiated BrAC recordings were obtained from 33,452 users. This was determined by the numbers available from the dataset provided by BACtrack.
Data exclusions	None
Replication	The accuracy of the machine learning algorithm developed in a training test set was then validated in a separate group of participants and subsequently tested in a third, separate, group.
Randomization	The cohort was randomly divided into into training (70%), validation (10%) and test sets (20%).
Blinding	Not applicable.

Reporting for specific materials, systems and methods

We require information from authors about some types of materials, experimental systems and methods used in many studies. Here, indicate whether each material, system or method listed is relevant to your study. If you are not sure if a list item applies to your research, read the appropriate section before selecting a response.

Materials & experimental systems

n/a	Involved in the study	
☒	☐	Antibodies
☒	☐	Eukaryotic cell lines
☒	☐	Palaeontology and archaeology
☒	☐	Animals and other organisms
☐	☒	Human research participants
☒	☐	Clinical data
☒	☐	Dual use research of concern

Methods

n/a	Involved in the study	
☒	☐	ChIP-seq
☒	☐	Flow cytometry
☒	☐	MRI-based neuroimaging

Human research participants

Policy information about studies involving human research participants

Population characteristics	Sex, age, and race were not available in this dataset. Geolocation was available, with approximately 50% of subjects residing in the US (country of origin is detailed in Table 1).
Recruitment	This analyses leveraged de-identified data collected from Bluetooth-enabled breathalyzers.
Ethics oversight	The University of California, San Francisco, Internal Review Board

Note that full information on the approval of the study protocol must also be provided in the manuscript.
